# LEACH Protocol Evolution in WSN: A Review of Energy Consumption Optimization and Security Reinforcement

**DOI:** 10.3390/s26072272

**Published:** 2026-04-07

**Authors:** Aijia Chu, Tianning Zhang, Chengyi Wang

**Affiliations:** 1 College of Science, Jiangsu University of Science and Technology, Zhenjiang 212100, Chinacywang@just.edu.cn (C.W.); 2 College of Computer Science, Jiangsu University of Science and Technology, Zhenjiang 212100, China

**Keywords:** LEACH protocol, wireless sensor network, energy consumption optimization, security enhancement

## Abstract

As a foundational protocol in wireless sensor networks (WSNs), LEACH has long contended with the dual challenges of energy load balancing and security defense. To clarify the protocol’s evolutionary trajectory within the modern IoT context, this paper presents a systematic review and restructuring of LEACH’s optimization mechanisms. The core contributions of this study are threefold: First, it establishes a taxonomy for energy optimization in LEACH. It provides an in-depth analysis of how intelligent algorithms—such as fuzzy logic and meta-heuristics—reshape cluster head election and data transmission paths in heterogeneous network environments, thereby resolving the inherent blindness of traditional mechanisms. Second, it elucidates the evolutionary patterns of LEACH security mechanisms. The paper details the transition of defense strategies from early static encryption and authentication to dynamic countermeasure mechanisms, offering a clear framework for understanding the protocol’s defensive boundaries. Finally, addressing the bottleneck where high security levels often incur high energy costs, the paper explores the feasibility of incorporating zero-trust architecture (ZTA) into WSNs within the future outlook section. This discussion aims to provide a new theoretical perspective for future research on balancing enhanced defense capabilities with energy efficiency.

## 1. Introduction

Wireless sensor networks (WSNs) serve as the foundational sensing layer for the Internet of Things (IoT), finding extensive application in domains such as military reconnaissance, environmental monitoring, and healthcare. These networks are typically composed of a vast number of resource-constrained micro-nodes tasked with the dual responsibilities of environmental sensing and data transmission. However, limited battery capacity remains a critical constraint governing the overall network lifespan. Particularly in traditional multi-hop flat routing paradigms, nodes located near the base station (BS) act as data forwarding “hubs” and often deplete their energy prematurely, leading to the “energy hole” phenomenon or even network paralysis. Such degradation in quality of service (QoS) arising from uneven load distribution has made the design of efficient routing protocols a focal point of research within the field.

To address these dilemmas, hierarchical routing architectures based on clustering have emerged as a dominant solution, distinguished by their superior scalability and energy efficiency [1]. In contrast to flat routing paradigms, where all nodes are functionally homogeneous, hierarchical routing typically employs a clustering strategy to partition the network into distinct logical regions. By designating cluster head (CH) nodes to manage intra-cluster data aggregation and communication with the BS, this structure significantly alleviates the communication overhead on ordinary nodes and reduces global network traffic, thereby enhancing energy utilization efficiency [2].

As a seminal protocol in hierarchical routing, LEACH is designed as a self-organizing, adaptive clustering protocol for WSNs, with the primary objective of minimizing energy consumption and extending system longevity through optimized communication. Its operational mechanism essentially integrates three core strategies [3]. First is adaptive clustering, where nodes spontaneously organize into local clusters; member nodes need only transmit data to their proximal cluster head, avoiding direct long-range transmission to the BS. Second, given that CHs bear the heavier burden of data forwarding, the protocol introduces a randomized role rotation mechanism. Nodes autonomously elect themselves as CHs based on a specific probability in different rounds, dynamically distributing the high-energy load across the entire network to prevent the premature depletion of any single node. Finally, the protocol employs data fusion strategies. CHs utilize the spatial correlation of data to perform local processing and compression. This reduces the volume of backhaul data and—given that communication is typically more energy-intensive than computation—further lowers the aggregate energy consumption of the network.

**Figure 1:**
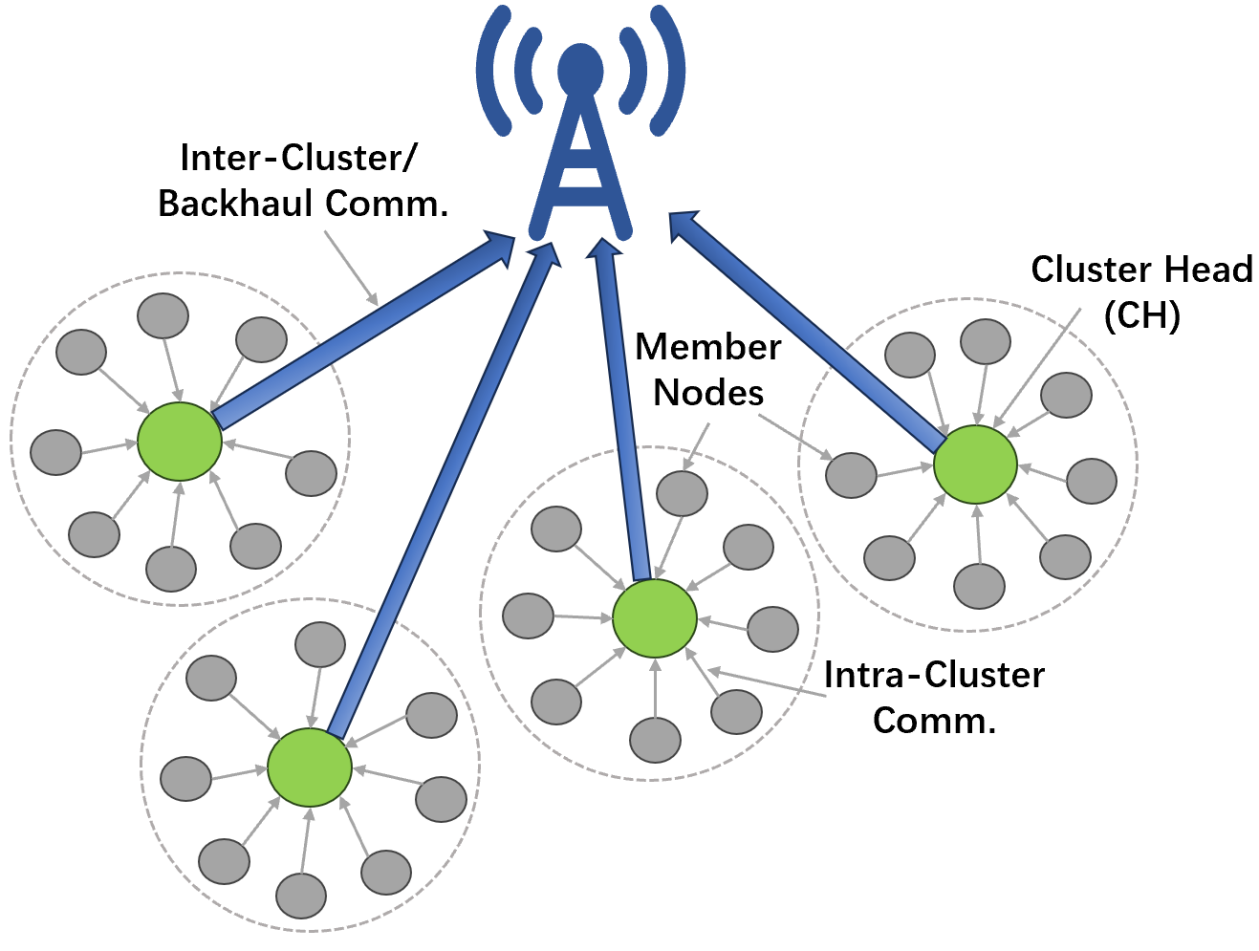
Architecture of the classical LEACH protocol.

However, while LEACH is theoretically groundbreaking, inherent challenges have become apparent when deployed in complex, real-world scenarios. First, the cluster head election mechanism suffers from stochastic limitations. Since the election relies solely on a probabilistic model without accounting for the residual energy of nodes [4], nodes with low energy reserves may still be elected as CHs, accelerating their failure. Furthermore, this purely probabilistic approach fails to guarantee a uniform geographical distribution of CHs, often leading to localized load imbalances. Second, the scalability of the single-hop communication architecture is restricted. The protocol mandates that all CHs communicate directly with the BS (Figure 1), ignoring the severe signal attenuation associated with long-distance transmission. For nodes at the network edge, this communication mode causes energy consumption to escalate drastically, severely limiting the protocol’s potential in large-scale network applications [3].

As shown in Figure 1, cluster heads located far from the base station (BS) face the risk of premature failure due to undertaking long-distance single-hop transmission tasks, while the randomized election mechanism further exacerbates the unevenness of cluster distribution. These two core defects have propelled extensive research on energy optimization in the subsequent two decades. Meanwhile, the original design of LEACH placed excessive emphasis on energy efficiency at the expense of security. Since this protocol is constrained by the inherent resource limitations, channel openness, and physically exposed environment of WSNs [5], and at the same time lacks necessary authentication and defense mechanisms, it appears extremely vulnerable when confronting inherent threats such as the openness of wireless channels and the physical exposure of nodes, rendering it highly susceptible to malicious attacks like HELLO flooding and selective forwarding [6].

In view of this, this paper aims to conduct a systematic review of the LEACH protocol and its subsequent improved variants. We will focus on evolutionary strategies within the two dimensions of energy efficiency enhancement and security defense. First, we provide an in-depth analysis of energy optimization schemes targeting cluster head election and data transmission paths. Second, we summarize security enhancement mechanisms aimed at strengthening protocol security and resisting external attacks. Furthermore, we explore comprehensive improvement models for the collaborative optimization of energy consumption and security. Finally, based on existing research bottlenecks, we discuss future technological trends.

## 2. Related Surveys and Research Methodology

### 2.1. Related Surveys and Motivation

Before delving into the specific technical optimizations of the LEACH protocol, it is essential to contextualize this study within the existing literature. In response to the rapid development of routing technologies, Al-Karaki N. and Kamal E. [7] systematically sorted out the core challenges of WSN routing design in their review, dividing the existing protocols into flat, hierarchical, and location-based categories. Focusing more specifically on the field of cluster routing, Yadav K. and Mishra R. [8] deeply analyzed the energy efficiency expansion mechanisms of LEACH and its improved versions, while also summarizing the preliminary security authentication algorithms.

However, most previous reviews dedicated to LEACH have tended to focus on specific, single dimensions of the protocol. In terms of energy efficiency optimization, Daanoune I. et al. [9] focused on comparing the performance of subsequent improved algorithms in extending network lifetime, whereas Patel D.K. et al. [10] specifically evaluated the impact of node density and cluster head ratio on protocol scalability. From a security perspective, Rahayu T.M. et al. [11] analyzed the defense mechanisms of SLEACH, Armor-LEACH, and other variants in detail. Furthermore, Ratnakumari P. and Rao M.K. [12] discussed the combined framework of energy efficiency and secure routing, though the scope of their discussion remained relatively limited.

**Figure 2:**
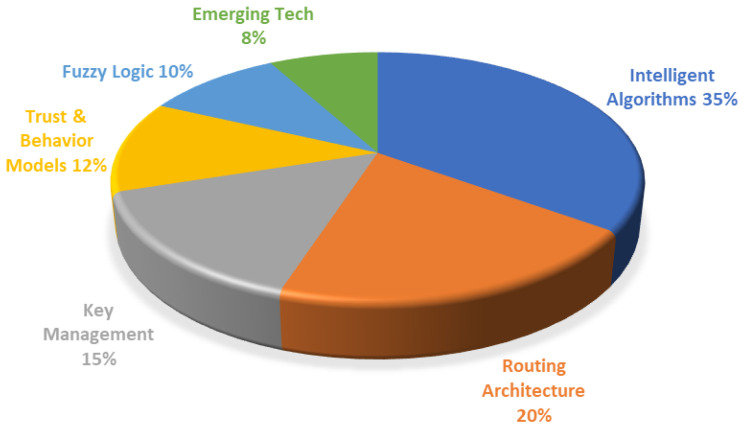
Statistical distribution diagram of the literature reviewed in this article.

Unlike the above reviews that focus on localized characteristics, this paper establishes an overall, comprehensive classification system covering the entire evolutionary pedigree of LEACH—spanning from foundational energy optimization to modern dynamic security. As illustrated by the literature statistics in Figure 2, although intelligent algorithms (35%) and routing architectures (20%) still dominate, reflecting the academic community’s continued attention to energy efficiency, security enhancements and emerging technologies have rapidly emerged as crucial research frontiers. Therefore, this review aims to seamlessly bridge the gap between traditional energy optimization and modern security defense, providing a holistic reference for the future design of WSN clustering protocols.

### 2.2. Paper Selection Method

To ensure the transparency and reproducibility of this review, we adopted a structured approach for literature search, screening, and thematic categorization. The literature search covered well-known academic databases, including IEEE Xplore, ScienceDirect, Elsevier, SpringerLink, and MDPI. The search strategy combined the main keywords: “LEACH protocol”, “wireless sensor networks”, “cluster head election”, “energy consumption optimization”, and “WSN security defense”. The time frame for retrieving literature is set from the inception of the LEACH protocol (2000) to early 2024, with a particular focus on the latest developments between 2018 and 2024. At the same time, we also used websites like Litmaps, Connected Papers, and ResearchRabbit to identify literature related to the original article on the LEACH protocol.

To maintain the high quality and relevance of this review, we adopted strict inclusion and exclusion criteria. Inclusion criteria: (1) peer-reviewed journal articles and high-quality conference papers written in English; (2) research proposing clear algorithmic or architectural improvements to the LEACH protocol; and (3) papers providing clear quantitative performance evaluations, such as energy consumption, network lifespan, or threat detection rate. Exclusion criteria: (1) preliminary abstracts of repetitive studies or those lacking detailed methods, and (2) research that relies solely on theoretical assumptions without simulation or empirical validation. Based on this rigorous screening process, approximately 120 core papers were selected for in-depth analysis.

### 2.3. The Origin and Construction of Taxonomy

**Figure 3:**
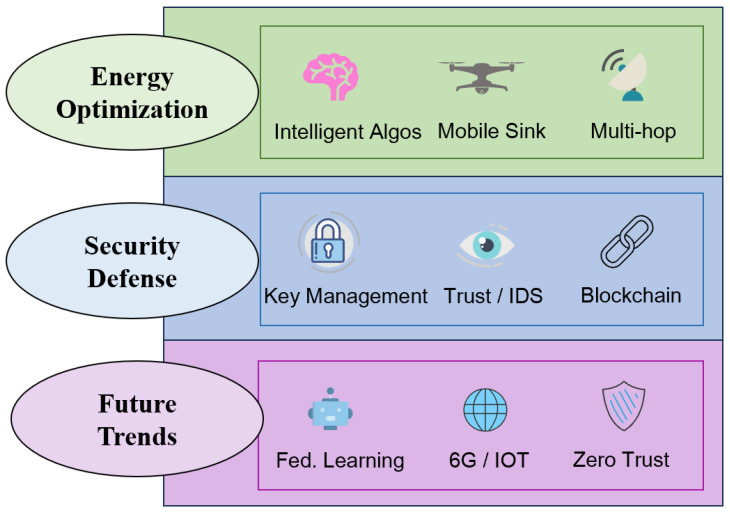
The evolution roadmap of the LEACH protocol.

The taxonomy presented in this paper was developed and constructed by the authors based on a comprehensive review of the selected literature. We did not use any existing classification systems; instead, we used a thematic synthesis approach. By extracting the main optimization objectives from the filtered literature, we observed a clear evolution from early energy-centric improvements to modern advancements driven by intelligence and enhanced security awareness. Therefore, we divide the progress into three main hierarchical pillars. To clearly outline this research environment, Figure 3 presents the overall roadmap of the evolution of the LEACH protocol.

**Figure 4:**
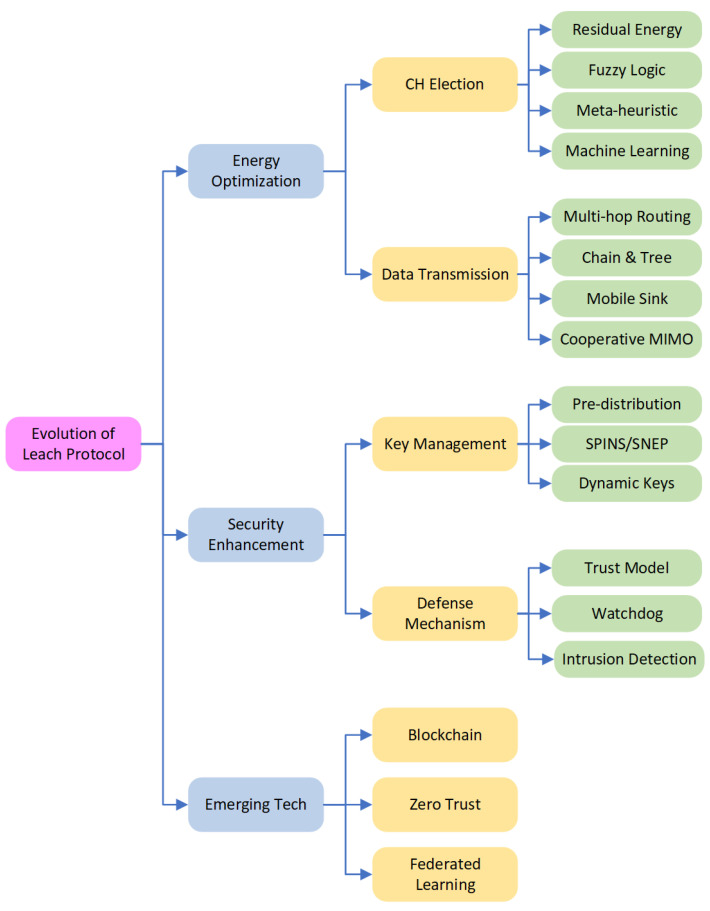
The taxonomy of LEACH protocol evolution.

According to our original framework design, the top layer highlights energy optimization strategies, covering everything from intelligent algorithms for electing cluster heads to multi-hop routing. The middle layer depicts the transition from basic key management to advanced trust models and blockchain integration for security defense. Finally, from the bottom layer, we focus on future trends, emphasizing the integration of federated learning, 6G/IoT, and zero-trust architecture. Based on this foundational roadmap, we further categorized the specific technical methods and algorithms used in the literature. Figure 4 further presents a comprehensive and detailed classification tree of these evolutionary paths.

This structured approach not only elucidates the historical trajectory of LEACH but also serves as the structural backbone for the subsequent chapters of this review, ensuring a balance between energy mechanisms and security defenses, as well as a coherent writing logic.

## 3. Optimization of Cluster Head Election Mechanism in Terms of Energy Consumption

**Figure 5:**
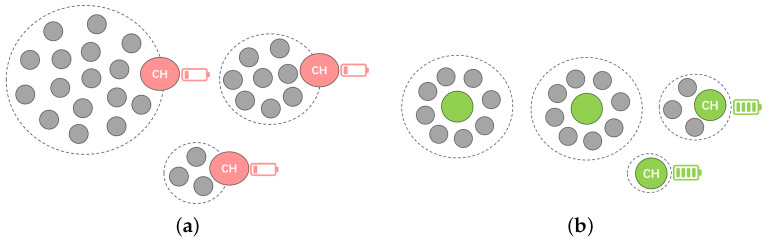
Comparison of cluster head election optimization mechanisms: (**a**) random election (LEACH); (**b**) intelligent optimization election.

To address the issues of uneven cluster distribution and imbalanced energy consumption resulting from the random selection of cluster heads in the LEACH protocol, researchers have proposed various optimization algorithms. Figure 5 intuitively compares the inefficient topology (a) that may be generated by the LEACH random election with the balanced topology (b) formed after optimization by intelligent algorithms.In the figure, the grey balls represent ordinary sensor nodes, and the dashed circles indicate the boundaries of the formed clusters.

As shown in Figure 5b, the main optimization goal during the election phase is to make the distribution of CHs more uniform and the cluster structure more compact, thereby reducing the total energy consumption of intra-cluster communication. In order to systematically explore the various mechanisms developed to achieve this goal, the remainder of this chapter is divided into three main categories. The first subsection reviews classic building improvements, including residual energy integration and multi-layer structures. The second subsection discusses the application of intelligent optimization and decision models, with a particular focus on heuristic methods and machine learning. Finally, the third section discusses the CH election model enhanced by fuzzy logic systems and game theory.

### 3.1. Classic Improvements

In response to the limitations of the original LEACH protocol regarding randomness, energy overhead, and scalability, subsequent research has focused on improvements across four key dimensions: introducing energy parameters, optimizing replacement mechanisms, constructing hierarchical structures, and refining election logic.

#### 3.1.1. Election with Residual Energy

The original LEACH protocol relies primarily on random probability for cluster head election, neglecting the current energy status of nodes. To rectify this deficiency, Ma. X. and Yu. X. [13] proposed the LEACH-ERE protocol. By modifying the calculation formula for the election threshold T(n) to correlate with the node’s residual energy, they increased the probability of high-energy nodes being elected. Building on this, M. J. Handy et al. [14] proposed the deterministic LEACH-DCHS protocol. Within a distributed decision-making framework, this protocol incorporates residual energy and the number of consecutive rounds a node has not been elected into the probability formula. By dynamically adjusting the threshold, it enhances the determinism of the election process and effectively extends the network lifetime.

#### 3.1.2. Cluster Head Replacement Based on Threshold

To mitigate the significant overhead caused by periodic cluster reorganization, J. Hong et al. [15] proposed the T-LEACH protocol. Abandoning the mechanism of mandatory cluster head replacement in every round, this protocol introduces an energy-based threshold: as long as the current cluster head’s energy remains above a preset level, it continues to serve even if the rotation period is reached. This on-demand replacement strategy significantly reduces unnecessary election processes and control signaling transmission, thereby lowering the overall energy consumption of the network.

#### 3.1.3. Multi-Level Hierarchical Structure

Targeting large-scale or high-density network scenarios, V. Loscri et al. [16] proposed the TL-LEACH protocol, which constructs a dual-layer structure comprising primary and secondary cluster heads to reduce long-distance data transmission through multi-level processing. Similarly, the LCH protocol by Y. Wang et al. [17] divides the network into multiple layers based on hop count to the base station. By forwarding data upward layer-by-layer and combining this with intra-layer rotation mechanisms, it achieves robust scalability. To ensure the stable operation of such hierarchical structures, M. Tong et al. [18] proposed the LEACH-B protocol. Furthermore, the energy-prioritization mechanism proposed by R. Ngangbam et al. [19] in EG-LEACH can be integrated into the election processes at various levels of these multi-level structures to further optimize system energy efficiency and longevity.

#### 3.1.4. Other Optimization and Improvement

Beyond the directions mentioned above, some researchers have reconfigured the underlying logic of the election process. Hu Junping et al. [20] proposed TB-LEACH, which replaces probabilistic election with a time-based competition mechanism. By utilizing random timers, it achieves adaptive control over the number of cluster heads without requiring global information. Heewook Shin et al. [21] proposed the COTS scheme, which completely eliminates the periodic cluster establishment phase. Instead, clusters are formed once during initialization, and roles are subsequently rotated among members based on a preset sequence, drastically reducing setup overhead. Additionally, H.M. Abdulsalam and L.K. Kamel [22] introduced W-LEACH, employing a weighted election mechanism where the base station calculates a comprehensive weight based on residual energy, neighbor density, and distance to ensure the selection of optimal cluster head nodes.

### 3.2. Cluster Head Election Based on an Intelligent Optimization Algorithm

In recent years, to address the limitations of traditional election mechanisms, researchers have introduced intelligent optimization algorithms into the cluster head election process for WSNs. These approaches fall primarily into two categories: heuristic algorithms and machine learning techniques.

#### 3.2.1. The Heuristic Algorithm

**a. Genetic algorithm (GA)**

Jyoti Bhola et al. [23] proposed a GA-based optimization of the LEACH protocol. The algorithm uses the fitness function of GA to select the node with higher residual energy as the cluster head (CH), which reduces the total energy consumption of the network and improves the packet delivery rate to a certain extent.

**b. Particle swarm optimization (PSO)**

Hu H. et al. [24] utilized an improved algorithm in the PFCRE protocol to implicitly perform cluster head election and load balancing without presetting cluster counts. Addressing the standard PSO’s tendency to get trapped in local optima, Haris M. and Nam H. [25] introduced a double exponential adaptive inertia weight (DEAI). This mechanism dynamically balances global exploration and local exploitation, effectively preventing premature convergence and enhancing energy efficiency.

**c. Bee colony algorithm**

Efati S. et al. [26] proposed a routing scheme combining the Markov model (MM) and artificial bee colony algorithm (ABC). In this scheme, MM first predicts and selects suitable CH candidate nodes based on the energy and location of the node history, and then the ABC algorithm performs a local search and approves and selects the final CH from the quality parameters, such as the energy and location of the candidate nodes. This method, which combines prediction and optimization, aims to improve load balancing, network quality of service (QoS), energy efficiency, and network life. However, the effectiveness of this method depends on the accuracy of the MM prediction and the local search ability of ABC.

**d. Elephant herding optimization**

M. Muthuselvi [27] proposed an energy-efficient stability-based clustering protocol, which adopted a phased optimization strategy. First, the improved fuzzy C-means algorithm is used to identify and initially select potential cluster heads by comprehensively considering multiple objective functions such as node residual energy, signal-to-interference-plus-noise ratio (SINR), load, and stability. Subsequently, a novel enhanced elephant herding optimization (EEHO) algorithm is introduced to establish the optimal communication path between the selected CHs and the base station (BS).

**e. Memory algorithm**

Wenfen Zhang et al. [28] proposed an adaptive clustering routing protocol based on a novel memory algorithm (MA). The protocol framework also divides nodes into three categories: cluster heads, cluster members, and free nodes, and uses a novel MA to optimize the clustering scheme. Unlike the methods that rely solely on random selection or simple heuristic rules, the MA aims to automatically determine the optimal number of clusters and select the appropriate CHs and free nodes by minimizing a multi-objective cost function that comprehensively considers the total energy consumption of the current round and the balance of residual energy between nodes.

**f. Other emerging algorithms**

Mistarihi M.Z. et al. [29] evaluated moth-flame optimization (MFO), whale optimization algorithm (WOA), and salp swarm algorithm (SSA) using a bi-objective function that accounts for intra-cluster distance and energy ratios, confirming the superior energy efficiency of MFO.

Addressing the limitations of specific algorithms, Liu Y. et al. [30] proposed the QCWOA algorithm to overcome the standard WOA’s tendency to get trapped in local optima. By integrating quantum optimization with hierarchical cloning operators, this approach optimizes clustering based on energy, distance, and transmission delay. Similarly, Di Jing [31] utilized an improved Harris hawks optimization (HHO) with a novel encoding mechanism to execute election and clustering simultaneously, thereby significantly reducing protocol overhead.

Furthermore, to achieve a more balanced load distribution, the ROA-MOCT protocol by Bourebia N.E.H. and Li C. [32] employed the raccoon optimization algorithm (ROA), incorporating node density and inter-cluster separation into its fitness function. Adopting a hybrid approach, the TSFC protocol by Yao Y. et al. [33] implemented a phased strategy: it first utilizes tuna swarm optimization (TSO) to improve structural compactness and subsequently leverages fuzzy logic to finalize cluster head selection based on residual energy and distance.

#### 3.2.2. Machine Learning and Data-Driven

Then, the improvement of the cluster head election mechanism by using machine learning and data-driven methods is summarized.

**a. K-means clustering**

K-means is frequently employed to structure network clustering. The KM-LEACH protocol by L. Ou et al. [34] performs k-means pre-clustering based on location before electing the highest-energy node within each cluster, effectively mitigating the stochastic nature of standard LEACH. Similarly, S.A.H. Al-Samhi et al. [35,36] adopted a coarse-to-fine strategy in the GSCK-TSP-MDC protocol, sequentially combining grid-based clustering with k-means to optimize load balancing and QoS.

**b. K-nearest neighbor (KNN)**

K-nearest neighbors (KNN) is predominantly employed to refine decision-making processes. Juwaied A. et al. [37] integrated KNN into established protocols for post-election optimization. By eliminating redundant cluster heads and reassigning member nodes based on proximity, this approach effectively minimizes communication energy dissipation.

In a different application, the KDL algorithm by Longla T.T. et al. [38] leverages KNN to assist DBSCAN clustering. Specifically, KNN is used to determine optimal clustering parameters and reassign outlier “noise points” to neighboring clusters. This establishes a robust structural foundation for the subsequent election of cluster heads based on energy and distance metrics.

**c. Artificial neural network (ANN)**

Addressing the limitations of single-parameter reliance in traditional protocols, Hoque M.J. et al. [39] introduced the NCRDAP protocol. This approach features a specialized artificial neural network (ANN) model that utilizes node energy, position, and base station distance to directly predict cluster head eligibility. Furthermore, the protocol integrates k-means clustering to refine the candidate selection, thereby minimizing intra-cluster distances and ensuring a balanced distribution of energy consumption.

**d. Reinforcement learning (RL)**

At the same time, reinforcement learning (RL) has been introduced for dynamic decision-making. Lasso J. et al. [40] proposed LEACH-RLC, where a centralized agent determines re-election timing to reduce the energy overhead of fixed schedules. In a distributed setting, Sattibabu G. et al. [41] applied federated reinforcement learning (FRL), with clusters training local Q-learning models to select cluster heads using energy, distance, and security indicators.

### 3.3. Cluster Head Election Based on Advanced Logical and Mathematical Models

On the basis of the traditional LEACH protocol, the introduction of advanced logical and mathematical models, such as fuzzy logic and game theory, is a common strategy to improve the cluster head election and routing energy efficiency.

#### 3.3.1. Fuzzy Logic

To mitigate the stochastic limitations of LEACH, Mamdani fuzzy inference systems are extensively employed for node evaluation. Sharma S. and Mittal N. [42] integrated residual energy, base station distance, and node density to optimize cluster head election, while Al-Husain and Al-Suhail [43] further extended network lifetime by incorporating node centrality. Additionally, for heterogeneous mobile networks, Li M. et al. [44] combined fuzzy logic with Mean Shift clustering to effectively address energy imbalances induced by node mobility.

Subsequent research has prioritized the deep integration of fuzzy logic with specific clustering algorithms. Shah and Ahmed [45] utilized fuzzy K-means within the FK-means RA protocol to optimize cluster structure and load distribution based on energy and distance. Meanwhile, Dutta R. et al. [46] introduced the LEACH-DCHS protocol, which employs a fuzzy-derived “opportunity value” to guide election, effectively preventing the selection of low-energy nodes and enhancing system stability. Furthermore, to address the limitations of traditional Type-1 fuzzy systems in handling the high uncertainty of WSN parameters (e.g., energy, distance, and density), A.J. Yuste-Delgado et al. [47] introduced a guided clustering protocol based on an Interval Type-2 fuzzy logic system (IT2FLS). By leveraging the superior uncertainty modeling capabilities of IT2FLS, this approach achieves more effective energy balancing and a significantly extended network lifecycle compared to conventional methods.

#### 3.3.2. Game Theory

In order to solve the high energy consumption overhead caused by the fixed-cycle re-election of the cluster head (CH) in the LEACH protocol, researchers also introduced game theory to construct a better CH election model. For example, Ntabeni U et al. [48] proposed the MFG-LEACH protocol, which models the CH election process as a mean field game. In this model, nodes decide whether to become cluster heads (CHs) based on their own energy status and the average behavior of other nodes in the network, which replaces the random probability used in LEACH and helps achieve Nash equilibrium for stabilizing and balancing network energy consumption.

Different game-theoretic models have been applied to specific election scenarios. Ning L. et al. [49] employed a non-cooperative dynamic game model in the EDRP-GTDQN protocol, optimizing cluster head selection via a payoff function that integrates residual energy and centrality to simultaneously minimize energy consumption and delay. Conversely, the GTFR protocol by Gangwar S. et al. [50] adopts a cooperative game strategy. Building upon fuzzy C-Means (FCM) clustering, it selects cluster heads based on energy and intra-cluster distance, thereby ensuring efficient energy management and prolonged network lifetime.

### 3.4. Synthesis and Discussion

To synthesize the reviewed methods and provide a unified perspective, Table 1 presents a comprehensive comparative analysis of intelligent CH election protocols. Highlighting their intrinsic connections, we categorize the cutting-edge literature into three primary paradigms: (I) heuristic and swarm intelligence algorithms, (II) machine learning and data-driven methods (including deep and reinforcement learning), and (III) fuzzy logic systems and game theory.

For each reviewed scheme, the table highlights the core technique employed and the specific optimization parameters considered for CH eligibility. Furthermore, to explicitly demonstrate the effectiveness of these advanced methods and provide a rigorous comparison, the Key Mechanism and Metrics column details both the core operational logic and the quantitative performance gains—such as percentage improvements in network lifetime, energy reduction, and throughput enhancements—relative to classical baseline protocols like LEACH.

## 4. Data Transmission and Routing Path Optimization in Terms of Energy Consumption

**Figure 6:**
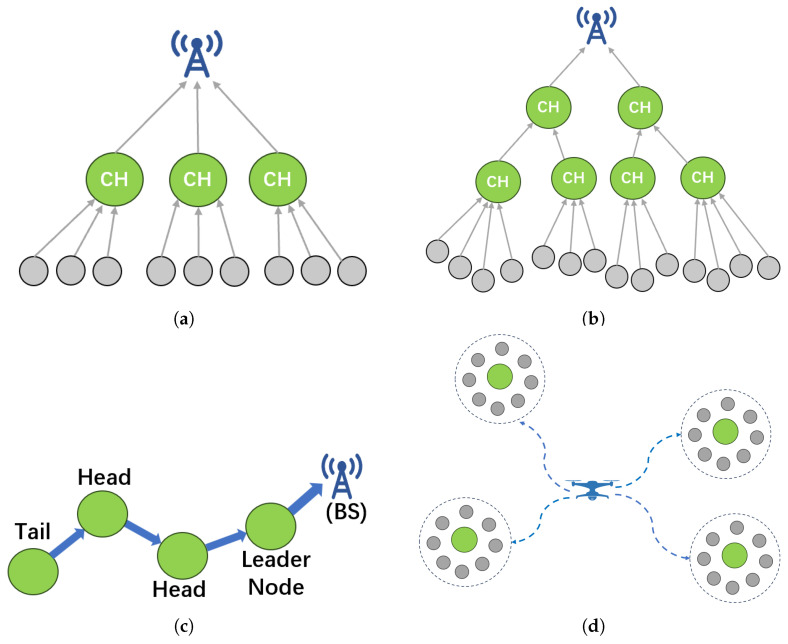
Comparison of data transmission and routing optimization strategies: (**a**) single-hop direct transmission; (**b**) multi-hop routing; (**c**) chain-based routing; (**d**) mobile aggregation.

While optimized CH election balances intra-cluster load, inefficient transmission paths to the base station (BS) remain a critical energy bottleneck, leading to premature CH depletion. Consequently, the research focus has expanded to routing path optimization to mitigate communication overhead and prolong network lifetime. Figure 6 contrasts LEACH’s single-hop transmission with evolved strategies, including multi-hop routing, chain routing, and mobile aggregation.

These different routing topologies aim to minimize the energy consumption of CHs by shortening the single transmission distance, avoiding hot spots, or introducing mobility. The following sections will review these strategies, respectively.

### 4.1. Chains and Tree Structures

To optimize the energy consumption of data transmission in the LEACH protocol, researchers have explored chain-based and tree-based routing paths.

#### 4.1.1. Chain Routing

Lindsey. S et al. [51] discussed the PEGASIS protocol in their review, which is an alternative to LEACH and organizes all nodes into a chain. Data is passed and fused hop-by-hop on the chain, and finally, only one leader node is responsible for sending the final data to the base station (BS) so as to eliminate the dynamic clustering overhead of LEACH and reduce the number of transmissions to BS to once per round.

#### 4.1.2. Tree Routing

Compared to chain topologies, tree routing is more widely applied to ameliorate the single-hop limitations of LEACH. Khamfroush H. et al. [52] proposed the TBR algorithm, which constructs a hierarchical routing tree based on residual energy and distance to mitigate energy consumption. Regarding spanning tree strategies, the DE-MST by Seelam K. et al. [53] utilizes a Minimum Spanning Tree (MST) to optimize inter-cluster transmission and load balancing, while WST-LEACH by Tang F. et al. [54] establishes optimal forwarding paths via a Weighted Spanning Tree (WST) based on energy and distance metrics. Additionally, LEACH-HPR by Li Han [55] adopts a flexible hybrid path strategy, dynamically switching between single-hop and multi-hop modes based on communication distance rather than constructing a complete tree.

### 4.2. Multi-Hop Routing

Given the bottlenecks and global knowledge requirements of single-chain structures, multi-path routing strategies offer enhanced flexibility and robustness by dispersing data traffic.

#### 4.2.1. Multi-Hop LEACH Variants

First, there are multi-hop LEACH protocol variants. Farooq M.O. et al. [56] introduced MR-LEACH, replacing the single-hop mechanism with a multi-hop architecture. By selecting optimal relay nodes based on residual energy and distance rather than direct long-distance transmission, this protocol significantly mitigates communication energy overhead.

#### 4.2.2. Multi-Hop Routing Combined with Multiple Intelligent Algorithms

The second is the multi-hop routing algorithm strategy combined with multiple intelligent algorithms. Wang C. et al. [57] proposed ICGA-LEACH, which utilizes a Chaotic Genetic Algorithm (CGA) to construct optimal multi-hop paths while simultaneously electing CHs. Wang Z. et al. [58] adopted a two-stage optimization in the NCRDAP protocol: initially employing Enhanced Snake Optimization (ESO) for election, followed by the Golden Jackal Optimization (GJO) algorithm to construct optimal routing paths.

In addition, regarding protocols previously discussed, distinct algorithms define their routing phases. Liu N. et al. [49] leveraged deep reinforcement learning (DQN) in EDRP-GTDQN to optimize paths for energy and delay following game-theoretic election; Li M. et al. [44] utilized the northern goshawk optimization (NGO) algorithm in NMSFRA specifically to generate forwarding paths for heterogeneous mobile networks. Additionally, Wang Z. et al. [59] applied Q-learning in the CRLM model for dynamic path optimization, while Altuwairiqi M. [60] implemented an improved honey badger algorithm (I-HBA) to enhance QoS and security.

#### 4.2.3. Two-Hop Routing

Finally, regarding the two-hop routing algorithm, Chen C. et al. [61] proposed the D2CRP protocol. Retaining the clustering concept, this approach incorporates a two-hop relay mechanism for both intra- and inter-cluster communication, aiming to alleviate the communication burden on CHs and ensure uniform load distribution.

### 4.3. Mobility Support

While multi-hop routing mitigates energy issues in static networks, the mobility of nodes or base stations (BS) in practical scenarios induces frequent topology changes, rendering static paths obsolete. Consequently, protocols must evolve to provide dynamic mobility support while maintaining energy efficiency.

#### 4.3.1. Mobile Node Support

To address connectivity loss caused by node mobility, Gu Y. [62] introduced the LEACH-M protocol, which enables nodes to dynamically switch to a new cluster head (CH) based on signal strength during transmission, thereby reducing packet loss without awaiting the next reconstruction round. To mitigate energy imbalances arising from frequent handovers, Qi W. et al. [63] proposed the ELBCFVD algorithm. This approach utilizes Voronoi diagrams for topology control, facilitating the rapid formation of balanced and stable clusters to minimize reconstruction frequency.

#### 4.3.2. Mobile Sink

Conversely, strategies utilizing mobile sinks (MSs) focus on optimizing data collection trajectories. Al-Sadoon M.E. et al. [64] developed the DTC-BR protocol, employing a two-layer clustering structure where the MS collects data exclusively from upper-tier Super CHs to conserve CH energy.

To further enhance trajectory efficiency, Gupta P.K. et al. [65] proposed the EEAC protocol, which elects specific “aggregation nodes” as relays, allowing the MS to visit only a subset of nodes. Building on this, Gantassi R. et al. [35] implemented a refined path planning strategy within the GSCK-TSP-MDC framework. Specifically, after clustering, the Traveling Salesman Problem (TSP) algorithm is applied to compute the optimal shortest tour for the Mobile Data Collector (MDC), thereby minimizing both delay and energy consumption during data retrieval.

### 4.4. Miscellaneous Optimizations

Beyond the primary topological and routing optimizations discussed above, researchers have also explored alternative avenues to enhance transmission efficiency, particularly focusing on heterogeneous environments, quality of service (QoS), and cooperative communication.

#### 4.4.1. Heterogeneous Network Support

Standard LEACH protocols often struggle with heterogeneous networks where nodes have varying initial energy levels. Addressing energy imbalance in such environments, Smaragdakis G. et al. [66] proposed the SEP protocol, which enhances stability by assigning differentiated election probabilities based on node energy levels. Building on this, Behera T.M. et al. [67] (I-SEP) and Kumar N. et al. [68] (TEEECH) introduced three-level energy models. These protocols utilize modified threshold formulas to accommodate complex heterogeneity, thereby significantly extending the network stability period.

#### 4.4.2. Specific Application Scenarios and QoS Considerations

Optimizations have also been tailored for specific monitoring contexts with strict quality of service (QoS) demands. For large-scale water monitoring, Zhong Y.C. et al. [69] proposed the EERCA protocol, which incorporates inter-cluster multi-hop routing and considers energy, distance, and density to mitigate excessive consumption. Similarly, for substation auxiliary services, Du Y. et al. [70] replaced the fixed-period rotation with a dynamic re-election mechanism based on residual energy thresholds, effectively meeting industrial requirements for high reliability and low energy consumption.

Recent research indicates a shift from solely minimizing energy consumption to balancing multidimensional QoS metrics. Advanced studies have explicitly included “data transmission delay” and “network stability” in their optimization goals. This shows how LEACH improvements have moved toward a full trade-off between energy efficiency, latency, and reliability.

#### 4.4.3. Cooperative Communication and MAC Layer Optimization

Finally, optimizations at the physical and MAC layers represent significant alternative strategies. Asaduzzaman and Kong [71] introduced EEC-LEACH, which employs cooperative MIMO technology. By organizing nodes into virtual MIMO systems for joint data transmission, this approach significantly lowers the power requirements for long-distance links, thereby extending network longevity.

Addressing the limitations of standard TDMA at the MAC layer, Thammawichai M. and Luangwilai T. [72] proposed 3D-TC-LEACH. This protocol integrates a coverage-based sleep scheduling mechanism that actively identifies and silences redundant nodes within a cluster, achieving energy savings superior to basic TDMA scheduling.

## 5. Security Threats and Static Cryptographic Defenses

Despite significant progress in energy optimization for LEACH, the inherent broadcast nature and unattended deployment of WSNs expose them to severe security vulnerabilities. For critical applications such as military reconnaissance or healthcare, energy efficiency alone is insufficient to guarantee system reliability. Malicious activities—ranging from node intrusion and eavesdropping to denial of service (DoS) attacks—not only compromise data confidentiality and integrity but also induce abnormal resource depletion, thereby rendering energy optimization strategies ineffective.

**Figure 7:**
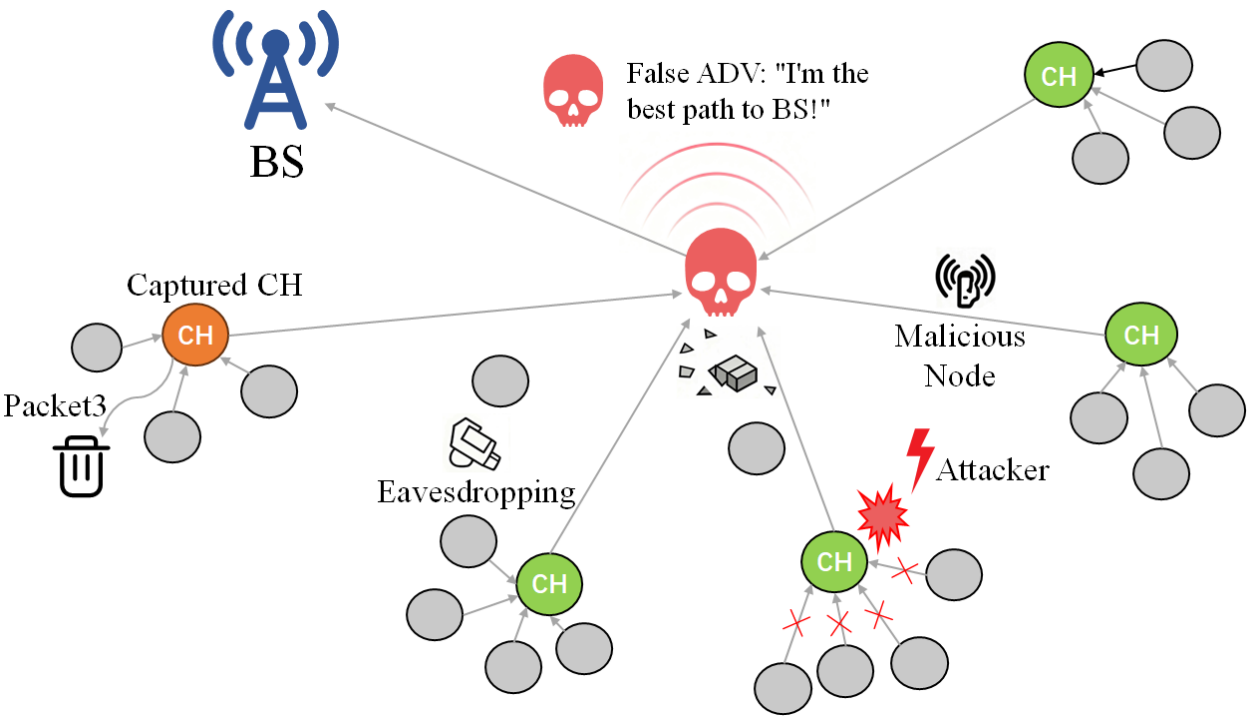
Schematic diagram of classic security threats in WSN.

To systematically analyze these vulnerabilities, we draw upon the comprehensive threat taxonomy established by Krishna et al. [73]. Based on their classification of network and perception layer attacks in IoT and sensor networks, Figure 7 illustrates several representative threats specifically tailored to the LEACH clustered architecture. As identified in the reference literature [73], these selected threats include sinkhole/blackhole attacks, eavesdropping, denial of service (DoS), and selective forwarding/packet dropping via captured cluster heads. This selection represents the most critical attack vectors that directly undermine the hierarchical routing topology of LEACH.Additionally, the red × symbols in the figure indicate disrupted or blocked communication links caused by the attacker.

**Figure 8:**
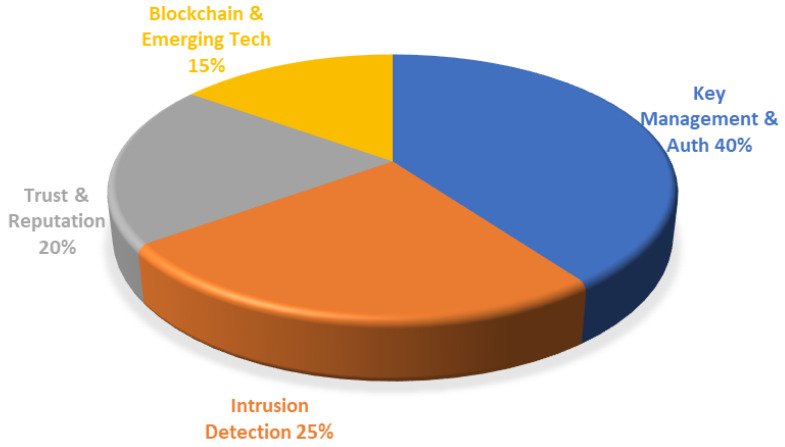
Distribution of security enhancement mechanisms in LEACH-based protocols.

Therefore, while optimizing the network energy consumption, building an efficient security mechanism to ensure the robustness of the protocol in a complex environment has become another core direction in this field. Figure 8 illustrates the distribution of security strategies in LEACH-based protocols. While key management (40%) remains foundational, the prevalence of intrusion detection (25%) and trust models (20%) signals a clear shift toward dynamic defense. Notably, the remaining 15% comprises emerging technologies like blockchain, reflecting a trend toward decentralized security architectures.

To comprehensively introduce these developments, this chapter will be divided into two main parts: the first section analyzes the basic security threats in wireless sensor networks (WSNs) and preliminary architectural extensions; the second section investigates static encryption methods, particularly key management and authentication. Building on these static foundations, the next chapter will dive into advanced dynamic defense mechanisms and emerging security technologies.

However, it must be acknowledged that implementing robust security mechanisms inherently brings additional computational and communication overhead. In resource-constrained wireless sensor networks (WSNs), this approach introduces a critical trade-off: while security protocols can prevent unauthorized access and data tampering, their energy consumption may inadvertently accelerate node depletion, which is a vulnerability that adversaries often exploit through depletion attacks. Therefore, we fully recognize that the energy impact of security mechanisms is a major issue. Although this chapter strictly focuses on the classification of threats and the architectural design of defense mechanisms, the critical assessment of their energy impact and the fundamental trade-off between security and network lifetime will be addressed separately in Section 7.

### 5.1. WSN Security Threat Analysis and Early Extensions

Due to the lack of intrinsic security mechanisms, LEACH is highly susceptible to various adversarial exploits. Ajay K.S. et al. [74] systematically analyzed denial of service (DoS) threats targeting the cluster head rotation mechanism, noting that adversaries can exploit HELLO floods or MAC layer interference to disrupt topology and deplete energy. These findings are further corroborated by Pathan Amjad S. et al. [75], who emphasized the severe impact of DoS and sinkhole attacks on WSN integrity.

To address general WSN security threats, Karlof C. et al. [76] proposed TinySec, a link-layer security architecture embedded within TinyOS. While it ensures message confidentiality, authenticity, and replay protection, the authors acknowledged its vulnerability to physical node capture and resource-exhausting DoS attacks. Specifically addressing LEACH vulnerabilities, Gangwar S. et al. [77] proposed the SLEACH protocol, incorporating SPINS and random key pre-distribution. While effective in preventing external intrusion and ensuring communication confidentiality via authentication, SLEACH, characteristic of early extensions, lacks robust defense mechanisms against internal attacks resulting from node capture.

### 5.2. Key Management and Authentication

Although early extensions like TinySec and SLEACH mitigated certain external attacks via encryption and authentication, they underscored a fundamental challenge: the security of encryption operations hinges on secure keys. Consequently, the efficient distribution, maintenance, and management of keys within resource-constrained networks, alongside reliable identity authentication, have emerged as central themes in subsequent security research.

#### 5.2.1. Key Management Schemes

**a. SPINS-based Schemes**

The SPINS protocol suite proposed by Perrig A. et al. [78], comprising SNEP for confidentiality and μTESLA for authenticated broadcasting, serves as a cornerstone for securing clustering protocols. Leveraging this, Ferreira A.C. et al. [79] developed the SecLEACH protocol. It employs μTESLA to secure CH identity and routing broadcasts while utilizing SNEP to guarantee the confidentiality and integrity of intra-cluster communications, effectively extending the security perimeter of LEACH.

**b. Random Key Pre-distribution**

Random key pre-distribution represents a prevalent early approach for enhancing LEACH. Oliveira L.B. et al. [80,81], in their comprehensive work on SecLEACH, demonstrated the mechanism’s applicability to dynamic clustering. Their protocol integrates SPINS with symmetric keys to ensure confidentiality and freshness without compromising the dynamic nature of LEACH. Similarly, Zhang K. et al. [82] proposed RLEACH, which adopts an improved Random Pairwise Key (RPK) scheme as a lightweight method to achieve efficient intra-cluster key management with reduced energy overhead.

**c. Hierarchical and Pairwise Keys**

To further bolster security, researchers have adopted pairwise and hierarchical key structures. The SC-LEACH protocol by Jiangtao Wang et al. [83] combines pre-shared pairwise key distribution with a low-power election algorithm to balance security and efficiency. The MS-LEACH protocol by Mona El-Saadawy and Eman Shaaban [84] enhances S-LEACH by utilizing shared pairwise keys between CHs and members for improved data confidentiality. Regarding hierarchical management, Singh U.R. et al. [85] explored an AVL tree-based approach within a hexagonal topology, using CHs as vertices to achieve secure pre-shared key distribution.

**d. Modern Encryption and Key Management**

Recent advancements have shifted toward modern encryption techniques. Urooja S. et al. [86] proposed a hybrid scheme utilizing Elliptic Curve Cryptography (ECC) for efficient key exchange combined with AES for data encryption. In a novel approach, Aissani S. and Abbache B. [87] introduced SKMSI-CL, which employs steganography to conceal key information within ZigBee packet headers, thereby mitigating the energy cost of key distribution. Additionally, Abuhelaleh and Elleithy [88] designed a specialized key management module within the SOOAWSN architecture specifically tailored for dynamic protocols like LEACH.

#### 5.2.2. Identity Authentication Mechanism

To prevent unauthorized nodes from accessing, the identity authentication mechanism has undergone an evolution from centralized verification at base stations to multi-factor authentication.

**a. Base Station and Remote Authentication**

Ping Li and Lin Ning [89] introduced the BSATLEACH protocol based on TLEACH. By mandating the base station (BS) to comprehensively evaluate both node identity and behavioral trust, this approach balances security requirements with energy constraints. In a centralized clustering context, Zhang Hong-jun [90] designed a remote authentication protocol where cluster heads act as servers, verifying adjacent nodes via message payload exchange prior to cluster admission.

**b. Modern Authentication Schemes**

Recent advancements focus on multi-factor authentication for enhanced security. Marope M. [91] proposed BA-LEACHC, which integrates a two-factor authentication phase into LEACH-C to effectively preclude unauthorized node entry. Furthermore, Alsumayt A. et al. [92] developed a comprehensive four-factor authentication mechanism tailored for IoT and WSN environments, significantly elevating the network’s security assurance through multiple verification layers.

## 6. Dynamic Defense and Emerging Security Technologies

**Figure 9:**
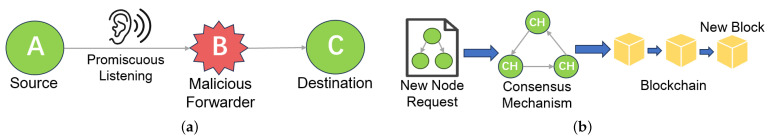
Working principle diagram of security defense mechanism: (**a**) watchdog detection mechanism; (**b**) blockchain technology.

While key management and authentication establish a robust primary defense against unauthorized access, these static measures are insufficient against internal threats such as node capture or compromise. Traditional security mechanisms are effective in dealing with known threats, but they are gradually exposing their limitations in the face of increasingly intelligent new attacks. Consequently, implementing a second layer of dynamic defense—specifically intrusion detection and attack mitigation—and actively exploring the application of emerging technologies such as machine learning and blockchain is essential for the real-time identification and isolation of malicious intra-network behaviors to build a more resilient and adaptive security system. Figure 9 illustrates two contrasting defense paradigms: (a) the “watchdog” mechanism based on real-time neighbor monitoring and (b) blockchain technology leveraging decentralized consensus for authentication.

### 6.1. Trust and Reputation Model

To address internal threats, introducing trust and reputation models has become a key means to make up for the deficiencies of encryption mechanisms.

**a. Trust-Aware Routing**

Regarding routing decisions, Fang W. et al. [93] proposed the LEACH-TM protocol, which incorporates hierarchical trust management into LEACH. By evaluating historical communication behaviors, it prioritizes nodes with both high trust and residual energy during the CH election to isolate malicious entities. Pu Gong et al. [94] introduced ETARP, innovatively applying “utility theory” to construct a utility function that weighs path credibility against energy efficiency.

Additionally, TADR-EAODV by Zhe Yang et al. [95] integrates AODV extensions with trust management to secure dynamic routing decisions. Osamy W. et al. [96] introduced SEACDSC, which fuses Discrete Sand Cat Swarm Optimization (DSCSO) with trust evaluation to globally optimize paths and evade malicious nodes.

**b. Reputation Model**

To enhance detection efficiency, Hongyu Yang et al. [97] proposed the MNDREL algorithm based on a reputation model. In this mechanism, nodes append reputation values to data packets. The Sink node then generates a suspicious list by analyzing source information and precisely identifies malicious nodes by calculating the ratio of their “suspicious value” to “credible value.”

### 6.2. Behavior-Based Detection

Beyond trust models, direct behavioral monitoring serves as an effective detection strategy.

**a. Watchdog Mechanism**

Rohbanian M.R. et al. [98] proposed Watchdog-LEACH, incorporating the classic watchdog mechanism into the clustered architecture. By designating specific nodes to monitor neighbor behaviors, this protocol establishes a robust defense against various attacks while preserving the inherent energy efficiency of LEACH.

**b. Intrusion Monitoring System**

Addressing active routing attacks such as packet dropping, flooding, and modification in multi-hop networks, Thivakaran T.K. and Sakthivel T.T. [99] introduced the GUARD framework. Its primary innovation, “limited directional watchdog selection”, minimizes energy overhead by activating only essential monitoring nodes to function as a lightweight IDS. Furthermore, the framework integrates non-cooperative game theory and fuzzy Q-learning to enhance the precision of malicious node identification.

### 6.3. Defense Against Specific Attacks

**a. Black Hole/Sinkhole/Wormhole Attacks**

Addressing destructive routing exploits, Vieira M.A. and Liu H. [100] proposed ARC-LEACH, which employs an “abnormal report cycle” to trigger CH rotation and BS coordination, effectively mitigating black hole attacks while balancing energy consumption. Similarly, to counter sinkhole and wormhole threats, Batool R. et al. [101] developed a secure routing protocol reliant on collaborative node mechanisms to enhance detection reliability.

**b. Denial of Service (DoS)**

Machine learning (ML) has become pivotal in mitigating DoS attacks. Khan Z.A. et al. [102] established the WSN-DS dataset as a benchmark for training Intrusion Detection Systems (IDS), validating the efficacy of ML algorithms. Building on this, Ahmad R. et al. [103] optimized classifier inputs via Feature Selection (FSA) and Mutual Clustering (MCA), improving DoS detection accuracy while maintaining network energy efficiency.

**c. Physical Layer Eavesdropping**

To secure physical layer communications in multi-hop scenarios, Hung H.D. et al. [104] analyzed a Cooperative Jamming (CJ) scheme. By utilizing energy-harvesting friendly jammers to actively degrade the channel quality for eavesdroppers, this approach ensures the confidentiality of inter-node transmissions.

### 6.4. Blockchain

#### 6.4.1. Secure Routing and Authentication

Due to the decentralized and tamper-proof characteristics of blockchain, researchers have introduced it into LEACH to solve the energy consumption and security defects of traditional authentication mechanisms. The SLEACH-PRO protocol proposed by Aljumaie G.S. and Alhakami W. [105] and the BSLEACH designed by Ajay K.S. et al. [74] both use blockchain to enhance the efficiency of identity authentication and data integrity.

Amjad S. et al. [75] combined the blockchain authentication mechanism with DDR-LEACH, requiring nodes to complete on-chain verification before participating in the network. For the 6G-IoMT scenario, Yuvarani R. et al. [106] further constructed a fusion framework of a lightweight blockchain layer and cluster head optimization algorithm to cope with the challenges of energy consumption and delay in a high-throughput environment.

#### 6.4.2. Blockchain Combined with Deep Learning and Real-Time Messaging Content Verification

In addition, the blockchain is also combined with deep learning for verification. Khan Z.A. et al. [107] proposed a dual-driven architecture that uses deep learning to optimize routing QoS while relying on blockchain to ensure transmission security. Khan A.U. et al. [108] integrated the real-time message content verification (RMCV) mechanism into ETD-LEACH and realized the accurate identification of malicious nodes through the collaboration of blockchain storage and real-time verification.

### 6.5. Machine Learning and Deep Learning

In order to make up for the neglect of security threats in traditional energy efficiency optimization, Aarti Sharma and Ankush Kansal proposed a series of secure routing schemes combining meta-heuristic algorithms and artificial neural networks (ANNs). In their study [109], the authors used the IEE-LEACH mechanism to select clusters, combined the firefly algorithm (FFA) to optimize node attributes, and finally used ANNs to identify and isolate malicious nodes. In their subsequent work [110], they further introduced the grasshopper optimization algorithm (GOA) into the routing optimization stage and also used ANN to achieve accurate classification of the “normal” or “malicious” state of the nodes, thereby constructing a transmission path that combines security and energy efficiency.

Building on this, Hajar Moudoud et al. [111] proposed the MAF-DRL framework, which integrates federated learning (FL) and multi-agent deep reinforcement learning (MADRL) to build a distributed intrusion detection system (IDS) with privacy protection capabilities.

To systematically synthesize the modern security enhancements discussed throughout this section, Table 2 provides a comprehensive comparative analysis. Rather than isolating these approaches, the table highlights how AI-driven models, trust architectures, and blockchain technology are collaboratively utilized to address complex vulnerabilities in modern LEACH variants.

## 7. Comprehensive Optimization and Future Prospects of LEACH Protocol

In summary, this paper has systematically reviewed the advancements in the LEACH protocol regarding energy efficiency and security enhancements. However, practical deployments reveal an inherent trade-off: heightened security often incurs additional energy overhead. Consequently, single-dimensional improvements are insufficient for dynamic application scenarios. The critical challenge, therefore, lies in achieving multi-objective synergistic optimization under resource constraints. The following section summarizes existing comprehensive optimization strategies and forecasts future research directions.

### 7.1. Collaborative Optimization of Energy Consumption and Security

#### 7.1.1. Early Integrated Protocols

Early research focused on integrated protocols to achieve synergy between energy efficiency and security. Banerjee P. and Jacobson D. [112] (GS-LEACH) utilized grid-based key pre-distribution to outperform traditional variants in both metrics. Similarly, SS-LEACH by Wu D. et al. [113] combined node self-localization with key strategies, enhancing routing security and network lifetime via dynamic multi-path CH chains.

Regarding architectural integration, Abuhelaleh M.A. et al. [114] introduced Armor-LEACH, a hybrid solution incorporating Time-Controlled Clustering Algorithms (TCCA) for low-power secure routing. Furthermore, Yang L. [115] improved LEACH-C by employing centralized BS management for election and encryption, effectively balancing network longevity and security overheads from a global perspective.

#### 7.1.2. Lightweight Security Protocol

To minimize the overhead of security mechanisms, Alshowkan M. et al. [116] proposed LS-LEACH, incorporating lightweight authentication to ensure data integrity and authenticity with minimal energy cost. Addressing the high energy consumption of earlier variants like MS-LEACH, Alselehibi Y.R. et al. [117] developed SLW-LEACH. This protocol optimizes the trade-off between security and power consumption, demonstrating superior performance in network lifetime and throughput.

#### 7.1.3. Secure Data Aggregation

In terms of data fusion optimization, Rahayu T.M. et al. [118] pointed out that the existing protocols lack the routing basis for supporting secure aggregation and then proposed the SRP protocol. The core of the scheme is to introduce the pairwise key (PWK) and cluster key (CK) mechanisms to ensure the confidentiality and integrity of the cluster head when collecting and fusing data, and effectively prevent malicious nodes from tampering with the aggregation results.

#### 7.1.4. Modern Comprehensive Security Scheme

With the introduction of intelligent technologies, modern research has begun to explore the deep integration of energy consumption and safety. The comprehensive schemes represented by NCRDAP [60], BA-LEACHC [91] and SEACDSC [96] show that the limitations of a single dimension can be effectively broken by incorporating improved meta-heuristic algorithms (such as the honey badger algorithm and sand cat swarm optimization) or a two-stage authentication mechanism into the routing decision. The work of Sharma A. et al. [110] further confirmed that the synergy of machine learning (ANN) and optimization algorithms can achieve accurate identification and isolation of malicious nodes while maintaining high energy efficiency of the network.

### 7.2. Future Development Trends

#### 7.2.1. Federated Learning

In response to the single point of failure risk and privacy leakage problems faced by the existing centralized optimization, the future evolution of the LEACH protocol is shifting toward distributed intelligence. The decentralized collaborative paradigm with federated learning (FL) as the core allows nodes to train models without sharing original sensing data. Recent studies [41,111,119] have successfully constructed a defense framework with privacy protection capabilities by combining FL with deep reinforcement learning or anti-interference mechanisms. This shift from “centralized data upload” to “local model training” will be the key path to balance data utility and user privacy in the future.

#### 7.2.2. 6G, IoT and Edge Computing

The application boundary of the LEACH protocol is deeply extended from the traditional static WSN to 6G, Internet of Things and edge computing. In response to the ultra-low latency and massive connection characteristics of the 6G era, recent studies [75,106] have begun to explore the integration of lightweight blockchain and cluster routing to meet the security needs of high-agile environments such as the Internet of Sensor Things (IoST) and the Internet of Medical Things (6G-IoMT). At the same time, the introduction of the edge computing paradigm [39,70] is reshaping the topological logic of the protocol, and the bandwidth bottleneck and calculation delay problems caused by a large amount of heterogeneous data are effectively solved by sinking the calculation tasks to the substation or routing edge nodes.

#### 7.2.3. Deep Integration of AL/ML/DL

In addition to the evolution of the architecture, the integration of AI and deep learning is also promoting the transformation of protocols from “static rules” to “autonomous decision-making”. Jurado-Lasso et al. [40] used reinforcement learning to replace fixed rounds, realizing the autonomous control of cluster reconstruction. Meanwhile, recent studies [28,49] have introduced deep reinforcement learning and dynamic optimization algorithms to achieve intelligent collaboration of cross-layer routing and QoS, providing a new paradigm for multi-objective optimization in complex network environments.

#### 7.2.4. Zero-Trust Security Architecture

In view of the security vulnerability caused by the traditional WSN relying on a static boundary and shared key, it has become an inevitable trend to introduce zero-trust architecture (ZTA) into the LEACH protocol. Based on the “never trust, always verify” concept proposed by Stephen Paul Marsh [120] and the architectural discussion of John Kindervag [121], the future evolution of ZTA-LEACH will be reconstructed around the three pillars of identity, trust, and data privacy.

First, the protocol needs to break through the static key limitation, use the physical unclonable function to generate a non-replicable hardware identity identifier for each node, and realize low-cost continuous verification under the architecture of “base station decision-making and cluster head execution”. On this basis, the election mechanism that only relies on the remaining energy is obviously insufficient. Introducing lightweight machine learning to quantify the node behavior reputation and incorporating the dynamic trust value into the election threshold are key to preventing malicious nodes from taking power. Finally, in order to solve the potential contradiction between the zero-trust “minimum privilege” principle and the data fusion demand, privacy protection schemes such as homomorphic encryption will become a breakthrough, enabling the cluster head to complete aggregation in the ciphertext state so as to better balance high security and network energy efficiency without obtaining decryption permission.

## 8. Conclusions

### 8.1. Summary

Since its introduction in 2000, the LEACH protocol has established a milestone position in WSN cluster routing. Looking back on its evolution over the past two decades, the relevant research has mainly focused on two core areas of energy consumption optimization and security enhancement.

To systematically synthesize the extensive and diverse literature discussed in this review, Table 3 provides a comprehensive, multidimensional cross-reference matrix of the evolution of the LEACH protocol. The table categorizes the core literature based on two main axes: the vertical axis represents specific optimization dimensions, such as cluster head election, data transmission, security defense, and comprehensive optimization. The horizontal axis depicts the core technologies employed, ranging from traditional routing architectures to modern artificial intelligence and emerging technologies.

Based on the literature distribution in Table 3, we can clearly observe the paradigm shifts within these two core areas:

First, in terms of energy consumption optimization, the research depth has clearly shifted from early single-parameter correction to intelligent decision-making. As the table illustrates, traditional routing methods have been largely superseded by intelligent algorithms and fuzzy logic. This indicates that researchers are increasingly relying on heuristic methods to solve complex energy balance problems, extending the optimization boundary from single cluster head election to multi-hop transmission path planning and mobile node support, thereby realizing a leap from shallow improvement to deep calculation fusion.

Second, the focus of security defense embodies a distinct evolution from passive encryption to active and multi-level defense. As shown in the table, early security protocols primarily relied on basic key management and static authentication. However, recent research heavily populates the columns of trust models, machine learning, and emerging technologies. This vividly reflects how defense mechanisms have developed from static cryptography to dynamic, behavior-based intrusion detection, ultimately incorporating blockchain and deep learning to build decentralized intelligent identity authentication.

Finally, the “comprehensive optimization” approach almost covers all technical fields. This proves that optimizing a single dimension is no longer sufficient to meet the needs of modern sensor networks. Current state-of-the-art protocols must integrate multiple technologies, such as combining intelligent algorithms with trust models or fusing machine learning with blockchain. This cross-technology integration provides a clear directional roadmap for designing secure and energy-efficient protocols for future IoT and 6G applications.

### 8.2. Challenges and Prospects

Although the improvement of the LEACH protocol has made great progress, the current research still faces three core challenges: first, the contradiction between algorithm complexity and node resources—high-performance intelligent algorithms are usually difficult to deploy on physical nodes with limited resources; second, the gap between simulation and reality—the idealized channel model is difficult to reflect the physical interference and high dynamic mobility in the real environment; third, the game between security and energy efficiency—how to minimize additional energy consumption while ensuring high-intensity defense is still an unsolved problem.

Looking ahead, the clustering and rotation mechanism of LEACH still has strong vitality in large-scale distributed scenarios such as IoT, 6G and edge computing. However, its security architecture needs to undergo fundamental changes, that is, from the traditional boundary defense to the “zero-trust” architecture [122]. The future evolution direction should be committed to reconstructing the cluster head into a micro-policy execution point, continuously verifying the traffic in the cluster, and combining distributed intelligent technologies such as federated learning to achieve deep integration of energy consumption, security, QoS and intelligent decision-making under the zero-trust framework.

## Figures and Tables

**Table 1:** Taxonomy and comparative analysis of intelligent cluster head election protocols.

Scheme	Core Technique	Optimization Parameters	Key Mechanism and Metrics
**I. Heuristic and Swarm Intelligence Algorithms**			
[23]	Genetic Algorithm	Input: Residual Energy	Replaces random probability with fitness screening. Energy −64.97%, throughput +17.44%, delay −45% vs. LEACH.
[24]	Improved PSO	Obj: Energy, Load Balance	Implicitly performs election and allocation. Energy −20.04%, throughput +31.04%, lifetime +26.39% vs. IBRE-LEACH.
[25]	PSO + Adaptive Weight	Input: Energy, Location	Introduces adaptive inertia weight. Network longevity +28% vs. LEACH, +10% vs. KM-PSO.
[29]	MFO/WOA/SSA	Obj: Intra-cluster Dist.	Benchmarks algorithms for energy efficiency. Lifetime +32.2% (2380 vs. 1800 rounds) vs. LEACH.
[30]	QCWOA	Obj: Energy, Delay	Combines quantum optimization with cloning. Lifetime +28.78% vs. O-LEACH.
[31]	Improved HHO	Input: Energy, Density	Novel encoding executes election and clustering. Energy −42.73%, throughput +12.7%, lifetime +34.2% vs. IBRE-LEACH.
[32]	Raccoon Opt.	Fit: Energy, Separation	Incorporates inter-cluster separation distance. Lifetime extended to 1060 rounds vs. 298 rounds in LEACH.
[33]	TSO + Fuzzy Logic	Input: Energy, Distance	TSO adjusts structure; Fuzzy Logic finalizes. Lifetime +33.5% vs. EE-LEACH.
**II. Machine Learning and Data-Driven Approaches**			
[34]	K-Means	Input: Location, Energy	Location-based K-Means pre-clustering. Lifetime +20% (120 vs. 100 days), energy efficiency +18.75% vs. LEACH.
[35]	Grid + K-Means	Obj: QoS, Load Balancing	Grid partitioning followed by secondary K-Means. Latency reduced to 45.9 ms, throughput +136%, stability +286% vs. LEACH.
[37]	KNN Integration	Input: Neighbor Energy	Removes redundant CHs and reassigns members. Energy consumption reduced by 14.1% to 22.7% across various protocols.
[38]	KNN + DBSCAN	Input: Noise Points	Uses KNN to tune DBSCAN parameters. Lifetime +86.67% vs. LEACH, +73.33% vs. DBSCAN-KMeans.
[39]	ANN + K-Means	Input: Energy, Position	ANN predicts CH eligibility; K-Means refines. Lifetime +19–23%, throughput +20–30% vs. baselines like LEACH-C.
[40]	Reinforcement Learning	State: Network Energy	Centralized agent decides re-election timing. Lifetime (HND) extended to 1050 rounds vs. 950 rounds in LEACH-C.
[41]	Federated RL	State: Local Cluster Info	Distributed model training via local Q-learning. Alive nodes +55%, energy efficiency +15%, delay −30% vs. DQN.
**III. Fuzzy Logic Systems and Game Theory**			
[42]	Mamdani FLS	Input: Energy, Density	Evaluates node competence via fuzzy inference. Improves lifetime, energy consumption, and PDR vs. LEACH-MF.
[44]	Mean Shift + FLS	Input: Mobility, Energy	Dynamic election for heterogeneous networks. Lifetime +235 rounds, throughput +154.9% vs. LEACH.
[45]	Fuzzy K-means	Input: Centroid Dist.	Constructs soft clusters via centroid distance. Alive nodes +115%, throughput +17% vs. LEACH.
[46]	Fuzzy Logic	Output: Opportunity Value	Calculated value to prevent low-energy selection. Lifetime (HNA) +30.44% vs. LEACH.
[47]	Interval Type-2 FLS	Input: Uncertainty	Handles high parameter uncertainty. Lifetime +130% (2115 vs. 919 rounds) vs. LEACH.
[48]	Mean Field Game	Obj: Nash Equilibrium	Models election as a Mean Field Game. Lifetime (FND) +34.02%, HND +11.23% vs. BRE-LEACH.
[49]	Game Theory + DQN	Obj: Centrality, Energy, Delay	Integrates game theory for CH selection and DQN for routing. Improves network lifetime, energy efficiency, and throughput vs. baselines.

**Table 2:** Comparison of modern security protocols based on AI and emerging technologies.

Ref.	Core Technique	Addressed Issues	Key Mechanism and Metrics
**I. Trust and Reputation Models**			
[93]	LEACH-TM	Malicious CH Election	Integrates trust management with dynamic CH selection. Improves lifetime (FND/HND) vs. LEACH/LEACH-SWDN and avoids large-scale packet loss under attacks.
[94]	Utility Theory (ETARP)	Route Reliability	Uses utility functions to balance path credibility and energy. Energy cost −2.4∼20.5% vs. LTB-AODV with similar safety performance.
[95]	Trust + AODV (TADR)	Dynamic Routing	Integrates trust management with AODV. Average packet transfer rate +6∼17% vs. LEACH-TM/TAAO-SDTIM.
[96]	DSCSO + Trust Value	Malicious Node Avoidance	Combines Sand Cat Optimization with trust scores. Improves network stability, alive nodes, and energy efficiency vs. MG-LEACH/BAT-Based.
[97]	Reputation Analysis (MNDREL)	Malicious Node Detection	Calculates suspicious/credible ratios to isolate attackers. Higher detection rate and lower false alarm rate vs. FMATM/HRTM.
**II. Behavior-based Detection and Specific Attack Mitigation**			
[98]	Watchdog Mechanism	Behavioral Monitoring	Designates nodes to monitor neighbor forwarding behaviors. Energy overhead +2% vs. LEACH.
[99]	Fuzzy Q-learning + GT	Active Routing Attacks	Lightweight IDS using limited watchdog selection and fuzzy logic. Detection accuracy +16%, throughput +13.9% vs. EAACK-AODV.
[102]	ML Algorithms (WSN-DS)	DoS Detection	Validates ML efficacy in DoS detection via WSN-DS benchmark. Overall, DoS detection accuracy reaches 98.5% using ANN.
[103]	ML + WC Feature Selection	Detection Accuracy	Combines Water Cycle feature selection with Decision Tree. Detection accuracy reached 100% (using 1 feature) and lifetime +30% vs. LEACH.
[104]	Cooperative Jamming (CJ)	Eavesdropping	Employs energy-harvesting friendly jammers. Significantly reduces end-to-end Secrecy Outage Probability (SOP) vs. baselines without CJ.
**III. Blockchain and Decentralized Architectures**			
[105]	Blockchain (SLEACH-PRO)	Auth. Efficiency	Replaces traditional auth to fix flaws and reduce energy costs.Energy consumption −10.93% and delay −52.82% vs. LEACH-PRO.
[74]	Blockchain (BSLEACH)	Node Infiltration	Hybrid blockchain with PoA consensus to remove malicious CHs. Under 20-Sybil attacks, packet drops −85% and alive nodes +50% vs. LEACH.
[75]	Blockchain + DDR-LEACH	Access Control	Mandates on-chain node verification prior to network participation. Network lifetime reached 1500 rounds and +50% vs. LEACH.
[106]	Lightweight Blockchain	6G-IoMT Security	Integrates lightweight chain with CH optimization for high throughput. Authentication accuracy reached 99% and network lifetime +27% vs. LEACH.
**IV. Machine Learning and Hybrid Intelligent Systems**			
[107]	DL + Blockchain	QoS and Data Security	Dual-driven: DL optimizes QoS; blockchain secures transmission. QoS +10.6% vs. DRL-ER.
[108]	Blockchain + RMCV	Content Verification	Synergizes blockchain storage with Real-time Message Content Verification. Throughput +14% and packet drop ratio −60% vs. SLEACH.
[109]	IEE-LEACH + FFA + ANN	Malicious Node ID	Uses ANN and Firefly Algorithm to identify and exclude malicious nodes.Packet loss rate −83% and network lifetime +33% vs. LEACH.
[110]	GOA + ANN	Secure Routing	Integrates Grasshopper Opt. with ANN to classify node legitimacy. Detection rate reached 99.8% and energy consumption −27.8% vs. LEACH.
[111]	FL + MADRL	Privacy-Preserving IDS	Fuses Federated Learning with RL for private, distributed IDS. Detection accuracy reached 99% and training time −98.1% vs. Boosting.

**Table 3:** Summary of LEACH protocol evolution literature.

Optimization Dimension	Fuzzy Logic	Intelligent Algorithms	RL	Routing Arch.	Network Arch.	Key Mgmt.	Trust Model	Emerging Tech.
Cluster Head Election	[27,31,33,42,43,44,45,46,47,50]	[13,14,15,20,22,23,24,25,26,28,29,30,32,34,35,36,37,38,39,57,58,96,109]	[40,41,48,49]	–	[16,17,18,19,21,66,67,68]	–	[93,96]	[41,74,75,106]
Data Transmission and Routing	[24,31]	[35,44,57,58,59,60,110]	[49,59]	[51,52,53,54,55,56,61,77]	[44,62,63,64,65,69,70,71,72]	–	[94,95]	[107]
Key Management and Authentication	–	–	–	–	–	[5,77,79,80,81,82,83,84,116,117,118]	–	[74,75,92,105,106]
Intrusion Detection and Defense	–	[102,103,109,110]	–	–	[104]	–	[93,94,95,97,98,99,100,101]	[108,111,119]
Comprehensive Optimization	[43,45]	[49,60,96,110]	[49,111]	[60,61]	–	[112,113,114,115,116,117,118]	[93,96]	[74,75,105,106,107,108,111]

## Data Availability

No new data were created or analyzed in this study. Data sharing is not applicable to this article.

## Abbreviations

The following abbreviations are used in this manuscript:

WSN	Wireless Sensor Network
LEACH	Low-Energy Adaptive Clustering Hierarchy
IoT	Internet of Things
CH	Cluster Head
BS	Base Station
QoS	Quality of Service
MDC	Mobile Data Collector
ZTA	Zero-Trust Architecture
GA	Genetic Algorithm
PSO	Particle Swarm Optimization
ABC	Artificial Bee Colony
ML	Machine Learning
FL	Federated Learning
IDS	Intrusion Detection System
DoS	Denial of Service
RMCV	Real-time Message Content Verification
